# Osteogenic differentiation of follicular stem cells on nano-Saghez scaffold containing BMP2

**DOI:** 10.1186/s13018-019-1507-0

**Published:** 2019-12-16

**Authors:** Hananeh Bayat, Hassan Shahabinejad, Mohammad Bayat, Sadegh Shirian, Abdolreza Mohamadnia, Mohammadreza Alijani, Arash Godarzi, Pegah Shojaei, Sahar Shojaei, Abolfazl Shevidi, Naghmeh Bahrami

**Affiliations:** 1 Craniomaxillofacial Research Center, Tehran University of Medical Sciences, Tehran, Iran; 2 Department of Endodontics, Henry M Goldman School of Dental Medicine, Boston University, Boston, MA USA; 3 Oral and Maxillofacial Surgery Department, School of Dentistry, Tehran University of Medical Sciences, Tehran, Iran; 4 Department of Pathology, School of Veterinary Medicine, Shahrekord University, Shahrekord, Iran; 5 Shiraz Molecular Pathology Research Center, Dr Daneshbod Pathology Lab, Shiraz, Iran; 6 Shefa Neuroscience Research Center, Tehran, Iran; 7 Chronic Respiratory Diseases Research Center, National Research Institute of Tuberculosis and Lung Diseases (NRITLD), Shahid Beheshti University of Medical Sciences, Tehran, Iran; 8 Department of Biotechnology, School of Advanced Technologies in Medicine, Shahid Beheshti University of Medical Sciences, Tehran, Iran; 9 Department of Tissue Engineering, School of Advanced Technologies in Medicine, Fasa University of Medical Sciences, Fasa, Iran; 10 Department of Biochemistry, Shahid Chamran University, Ahvaz, Iran; 11 Gandi Hospital, Tehran, Iran; 12 Department of Tissue Engineering, School of Advanced Technologies in Medicine, Tehran University of Medical Sciences, Tehran, Iran

**Keywords:** Bone tissue engineering, Mesenchymal stem cells, BMP2, Scaffold

## Abstract

**Background:**

Bone tissue is one of the tissues that are capable of self-regeneration. However, bone self-regeneration is defeated in the case of broad lesion of bone structure. Isolated stem cells from wisdom tooth follicles can potentially differentiate into ectodermal and mesodermal cells. Saghez is a natural substance that has been extracted from *Pistacia terebinthus* with unique features, such as high temperature and mechanical stability, adhesive structure, biocompatibility, and anti-neoplastic properties.

**Methods:**

In this study, Saghez-encapsulated BMP2 was applied as a scaffold for wisdom tooth follicle stem cell differentiation into the osteocyte. A total of three wisdom tooth follicles were obtained for stem cell isolation. For verification of differentiation of the isolated stem cells into osteocyte and adipocyte, Oil Red and Alizarin staining were applied, respectively. Moreover, mesenchymal stem cells were distinguished by profiling their cell surface markers, includingCD73, CD90, CD44, and CD105, by flow cytometry. Saghez scaffold loaded with BMP2 factor was prepared using sol-gel method. Four experimental groups were considered in this study: cells seeded on BMP2 encapsulated in Saghez scaffold, Saghez scaffold, osteogenic medium, and DMEM medium.

**Results:**

Mechanical properties of Saghez scaffold, including tensile Young’s modulus, ultimate tensile stress, compression Young’s modulus, and complex shear modulus, were 19 MPa, 32 MPa, 0.42 MPa, and 0.9 MPa, respectively. The porosity of the scaffold was 70–140 μm, and the percentage of porosity was 75–98%. The results of flow cytometry studies indicated that CD44, CD73, CD90, and CD105 were positively expressed on the membrane of the tooth follicles’ stem cell. The results indicated that the rate of differentiation of the follicle stem cells into osteocyte was the highest in the Saghez-BMP2 scaffold containing differentiation medium groups. These findings were verified by morphological studies, osteoblast and osteocalcin gene and protein expression investigations, and alkaline phosphatase activity measurement. The highest osteopontin and osteocalcin genes expression levels (1.7 and 1.9) were seen in positive control, followed by DMEM + differentiation factor (1.5 and 1.6), scaffold + BMP2 (1.2 and 1.4), DMEM + stem cell (1 and 1) and scaffold (0.4 and 0.5), and negative control respectively.

**Conclusion:**

This study provides a novel system for differentiation of the stem cell into osteocytes. The results of this study suggest that loaded BMP2 in Saghez scaffold possibly acts as an osteocyte differentiator factor.

## Background

Tissue loss and organ failure are considered as major medical challenges all over the world. Annually, about 6.3 million cases of bone fracture occur in the USA, of which 550,000 cases require bone grafting [1].

Tissue engineering using relevant synthetic or natural scaffolds has been considered as a promising alternative to the natural bone grafts [2–6]. The chemical and physical properties of the scaffold directly affect cells proliferation [7]. It is essential that scaffolds maintain some features, such as pores for cell seeding and migration, ordered biodegradation simulated with tissue healing, and injectability for its transfusion with or without encapsulated cells to the body organs [8]. Basically, four types of polymer-based materials including synthetic, natural, hydro-gels, and composite polymers have been introduced for TE. The use of gum rosins has been shown as a precursor for advanced biomedical applications such as TE systems [9]. Gum rosin or Chios mastic gum extracts, locally named Saghez, is extracted via local procedure from *Pistacia terebinthus*, known commonly as turpentine tree. It is a species of *Pistacia*, native to Iran, and other regions such as the Mediterranean region, and Portugal to Greece and western and southeast Turkey. Saghez is a natural and biocompatible material that acts as an anti-inflammatory and anti-oxidative agent. These functions of Saghez are respectively correlated with TNFα and protein kinase C [10]. The combination of Saghez with chemotherapeutic agents induces apoptosis in squamous cell carcinomas [11]. Bone substitutes mainly involve three important biological properties including osteoconduction, osteoinduction, and osteogenesis. These biological capability of the graft materials is to differentiate the multipotent MSCs to bone-forming cells and induce osteoprogenitor cells and new bone formation [12]. Such biological ability has been discovered in osteogenic growth factors including platelet-derived growth factor (PDGF), transforming growth factor-β (TGF-β), insulin-like growth factor (IGF), fibroblast growth factor (FGF), and bone morphogenetic proteins (BMPs) such as BMP2 and BMP7 [13]. It has been found that BMP2 positively affects osteogenesis in bone fractures; therefore, it is widely applied in various scaffold encapsulations in order to deliver the scaffold to the fracture [14]. In recent years, application of different types of stem cells, including bone marrow stem cells (BMSCs), periodontal ligament progenitor cells (PDLSCs), and adipose-derived stem cells and MSCs, has been investigated for bone regeneration. Similarly to other stem cells, mesenchymal stem cells have some properties; for instance, they can differentiate into any other cell lines, preserve their morphological and functional properties, and reconstruct themselves during the life span of the organism [14]. Wisdom tooth follicles are a good source of potent stem cells, which can differentiate into osteocytes and adipocytes. In other words, these intact isolated stem cells are able to differentiate into all exodermal and ectodermal types of cells [14]. The aim of this study was to prepare Saghez scaffold loaded with BMP2 to investigate whether it could be a sufficient osteogenic differentiator to be used as a fabricated ECM for differentiation of wisdom tooth stem cells.

## Materials and methods

### Primary stem cell isolation and culture

Fresh wisdom tooth was obtained from three patients (age range of 15 to 30 years) via surgery. Patients signed an informed consent form prior to the surgery. The tooth was immediately transferred to the lab floating in Hanks’ medium. It was then washed with phosphate-buffered saline medium (PBS) containing antibiotic several times in order to digest the tissue. The isolated stem cells floated in Dulbecco’s modified Eagle medium that contained the floating isolated stem cells, and undigested tissue particles was passed through 40 μ and 70 μ filters. The medium was then centrifuged at 1500 rpm for 5 min. The isolated cells were finally seeded in a density of 10^5^ T75 culture flask and fed with DMEM medium containing 10% FCS.

### Differentiation of wisdom tooth follicle stem cells into osteoblast and adipocytes

To determine whether the isolated cells were stem cells, they were induced to differentiate into adipocyte and osteoblasts by specific cell culture media. The isolated cells were successfully differentiated into the bone-mentioned cells. Cells were seeded at a density of 20,000 cells per ml in 24-well plates in DMEMF12 containing 10% fetal bovine serum (FBS). The differentiation medium was added into the wells, 1 day after seeding the cells. The culture medium used for adipocyte differentiation which contained 50 mg/ml ascorbic acid three phosphates, 100 ng dexamethasone, and 50 μg/ml Indometacin. To differentiate the isolated stem cells into osteocytes, cells were treated with DMEM/F12 containing 50 μg/ml ascorbic acid three phosphates, 10 ng dexamethasone, and 10 mM beta-glycerol phosphate. The isolated stem cells were treated with these media for 14 days. The culture medium was refreshed three times weekly. Oil Red staining was used to determine the success of differentiating the isolated stem cells into adipocytes. Briefly, the differentiated cells were fixed and subsequently washed with 70% alcohol. Cells were then incubated with a coloring agent for 15–20 min and washed again with 70% alcohol [12].

Successful differentiation of the isolated stem cells into osteogenic cells were confirmed by Alizarin red staining. Osteogenic cells were washed three times with PBS prior to being fixed in 10% formaldehyde at room temperature. Cells were then stained with 1 ml Alizarin red color (pH = 4.1) for 20 min. The excess dye was removed by several washing steps using PBS before visualizing the cells under a light microscopy.

### Flow cytometry analysis

Flow cytometry analysis was applied to distinct mesenchymal and hematopoietic stem cells. After three passages, cells were trypsinized and used for the experiment at a density of 10^5^ cells per ml of PBS. The trypsinized cells in PBS were then treated with 5 μl CD44-FITC (Exbio/Czech), CD90-FITC (Exbio/Czech), CD45-FITC (Exbio/Czech), CD73-PE (Exbio/Czech), CD34-PE (Exbio/Czech), and CD105-PE (Exbio/Czech) antibodies. In this study, Mouse IgG1-FITC, Mouse IgG1-PE, and Mouse IgG2a-PE were used as the controls in separate tubes. Cells were then incubated in a dark room for 30 min prior to being centrifuged in 1 ml of washing buffer and were then centrifuged at 1500 rpm for 5 min. The cells were finally resuspended for flow cytometry detection (BD FACS Calibur using BD bioscience USA, CA—San Jose), and the results were analyzed using FlowJo 7.6.1 software.

### Fabrication and characterization of the scaffold

#### Preparation of micro and nanoporous scaffold

Saghez scaffolds were synthesized by hydrolysis-condensation (sol-gel) method. The first step of preparing the scaffold was dissolving Saghez (1.6 ml) in 15 ml of octane containing 0.2 M AOT and 0.04 M 1-HP by stirring the solution at high temperature. The solution was vortexed to become clear. A cross-linker agent, 60%mol divinyl sulfone (DVS) [15], was then poured into the solution and shaken at 2000–2200 rpm for an hour at room temperature. The solution was then filtered using Whatman papers (cutoff 8 μm) and prior to be added to acetone solution. Then, two-phase, acetone/isooctane was constituted. The organic phase which consists of acetone and isooctane was thrown out, and the precipitated materials were solved in acetone again and were repeated for three times. Subsequently, the air-dried gel was incubated at high temperature for calcination and was then encapsulated by BMP2 in the concentration of 30% using a double-emulsion method.

#### Morphological investigation

The micro-architecture of Saghez scaffold was investigated using scanning electron microscopy. The air-dried scaffolds were coated with gold sputtering and assessed at an accelerating voltage.

#### Mechanical stability

Mechanical behavior of the scaffold was studied by compression Young’s modulus, complex shear modulus, tensile Young’s modulus, and tensile stress.

#### Investigating in vitro drug release

A specific amount of the dried scaffolds was immersed in PBS solution with various pH values (7.4 and 6.5) at 37 °Ϲ under sink condition. In determined intervals, the amounts of the released drug were alternately measured using UV-Visible spectroscopy at 570 nm.

#### Cytotoxic effects of Saghez scaffold

The cytotoxicity of Saghez scaffolds was studied using two cell lines: NT2 (nerve progenitor cells) and HEK293T (human embryonic kidney cells). The cells were seeded in 96-well plates at a density of 3000 cells/well. After 72 h of being cultured on the scaffolds, the percentage of the live cells was determined using MTT assay (0.5 mg/ml of MTT powder).

#### Differentiation of the isolated stem cells into osteocytes

The isolated stem cells were cultured in four different conditions in order to investigate their differentiation into osteocytes. The cell culture conditions used for this purpose included cells seeded on the scaffold without BMP2 in osteogenic medium, cells seeded on the scaffold-BMP2 in osteogenic medium, cells cultured in DMEM/FBS medium, and cells cultured in osteogenic medium. After 2 weeks of being in culture, cell differentiation into osteocyte was investigated by morphological, gene, and protein expression studies.

#### Morphological survey

Differentiation of the isolated stem cells, which was seeded on the prepared scaffolds, into osteocytes was confirmed using scanning electron microscopy.

#### RNA extraction and quantitative real-time PCR (RT-qPCR)

Scaffolds were seeded with the differentiated stem cells at a density of 10^5^ in a 24-well plate. The expression levels of osteocalcin and osteopontin were studied by RT-PC. Total RNA was extracted using AccuPower® RocketScript™ RT PreMix kit. RNA concentration and purity were assessed by measuring 260/280 nm absorbance on a nanospectrophotometer. Isolated RNA with a 260/280 ratio of ~ 2 was used for further experiments. Isolated RNA was reverse-transcribed to cDNA using of the cDNA synthetic Kit (Takara, Japan). Gene expression was determined by real-time PCR using SYBR green. β-actin was chosen as a housekeeping gene and used as an internal comparator in parallel with the control sample (primer sequences and details Table 1). Relative gene expression was analyzed using StepOne software V2.0, and the baseline and threshold were set manually. RT-PCR data were analyzed using the ΔΔCt method.

**Table 1:** Details of the primers used in real-time PCR

Gene	Primer sequence	Product length (bp)	Accession number
Osteopontin	FW: CATCACCTGTGCCATACCAGTTA RV: TTGGAAGGGTCTGTGGGGCTA	149	(NM_000582.2)
Osteocalcin	FW: TCCTTTGGGGTTTGGCCTAC RV: CCAGCCTCCAGCACTGTTTA	148	(NM_199173.5)

*FW* forward, *RV* reverse, *bp* base pair

#### Western blot

Cultured stem cells were lysed in radioimmunoprecipitation assay (RIPA) buffer and proteins separated on a 10% SDS-PAGE and transferred onto a polyvinylidene difluoride (PVDF) membrane. The blot was incubated at 4 °C overnight with 0.2 μg/ml anti-rabbit osteopontin, anti-osteocalcin, and anti-beta actin antibodies in TBS-T buffer containing 2% BSA. The membrane was washed with TBS-T and incubated with 2 μg/ml polyclonal donkey to rabbit IgG conjugated to horseradish peroxidase (Abcam) in TBS-T buffer containing 2% BSA at RT for 60 min. Visualization was carried out using ECL reagents and developed on a film.

#### Alkaline phosphate activity

The cultured cells were fixed using citrate-acetone solution and treated with diazonium salt. The fixed cells were then washed with distillate water twice and stained by hematoxylin. The stained cells were visualized under a light microscope, and the results were analyzed using ImageJ software.

## Results

**Fig. 1:**
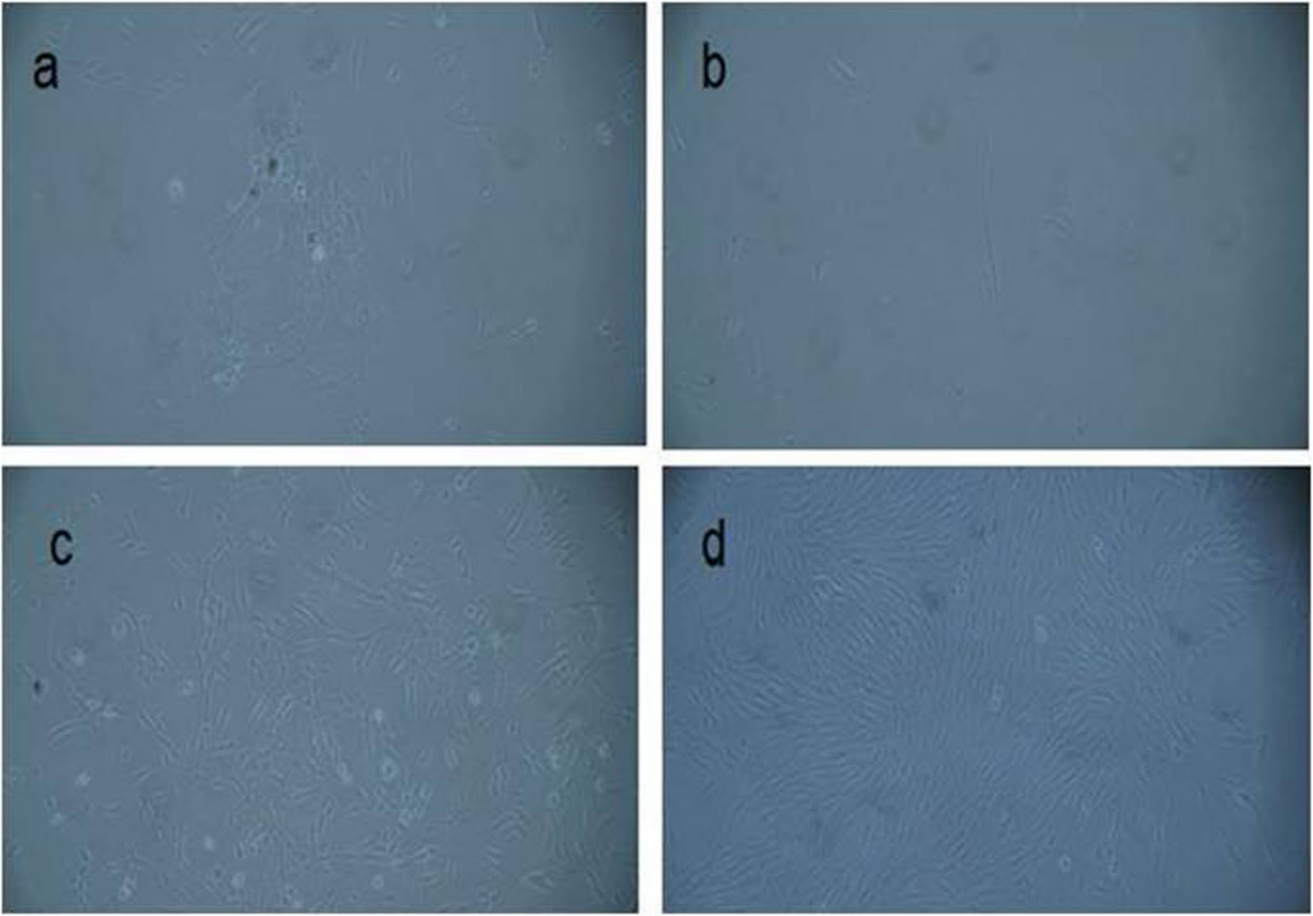
The isolated wisdom tooth follicle stem cells. Images were captured on days **a** 1, **b** 3, **c** 5, and **d** 7 post-seeding

The isolated wisdom tooth follicle stem cells on days 1 to 7 post-seeding is shown in Fig. 1.

### Differentiation of the wisdom tooth follicle stem cells into osteoblast and adipocytes

**Fig. 2:**
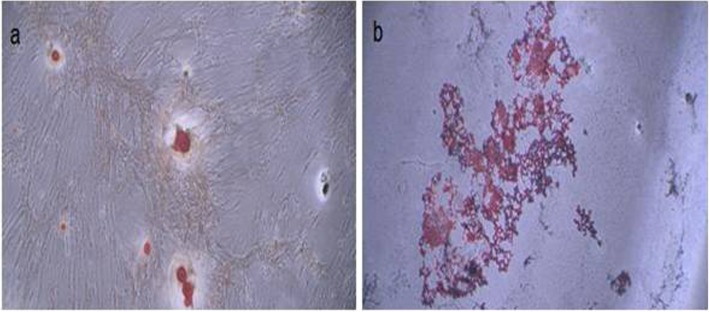
Differentiation of the wisdom tooth follicles stem cell into adipocytes and osteocytes. **a** Calcium precipitation and mineralized nodules in cells, which confirm osteocyte differentiation. **b** Lipid droplets in intracellular space which verify adipocyte formation

Treatment of the isolated stem cells with adipocyte and osteocyte differentiation media resulted in the differentiation of all the cells into the respective cell lines. Differentiation of the wisdom tooth follicle stem cell into adipocytes and osteocytes, which was respectively verified by Oil Red and Alizarin staining, is illustrated in Fig. 2. Precipitation of oil droplets in the ECM of adipocytes, which was detected by Oil Red staining, indicated that the stem cells were differentiated into adipocytes after treatment with specific a culture medium.

### Flow cytometry analysis

**Fig. 3:**
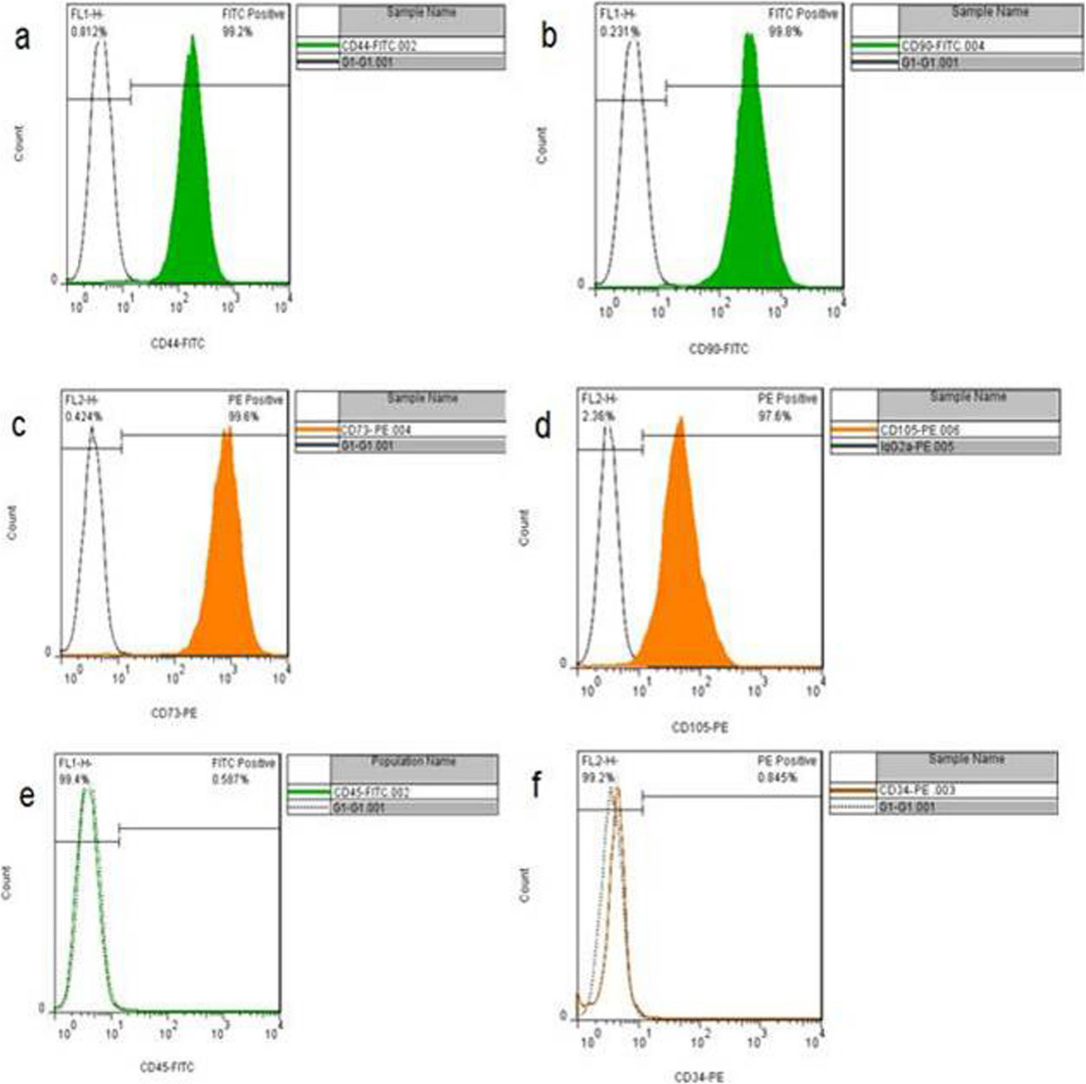
Percentages of expression of the mesenchymal markers. **a** 99.2% CD44, **b** 99.8% CD90, **c** 99.6% CD73, **d**97.6% CD105, **e** 0.587% CD45, and **f** 0.845% CD34

The results of flow cytometry studies indicated that CD44, CD73, CD90, and CD105 were positively expressed on the membrane of the stem cell. These markers are specific to the mesenchymal cell. However, CD34 and CD45, which are specific to a hematopoietic line, were not completely exposed (Fig. 3).

### Morphological investigation of the scaffold

**Fig. 4:**
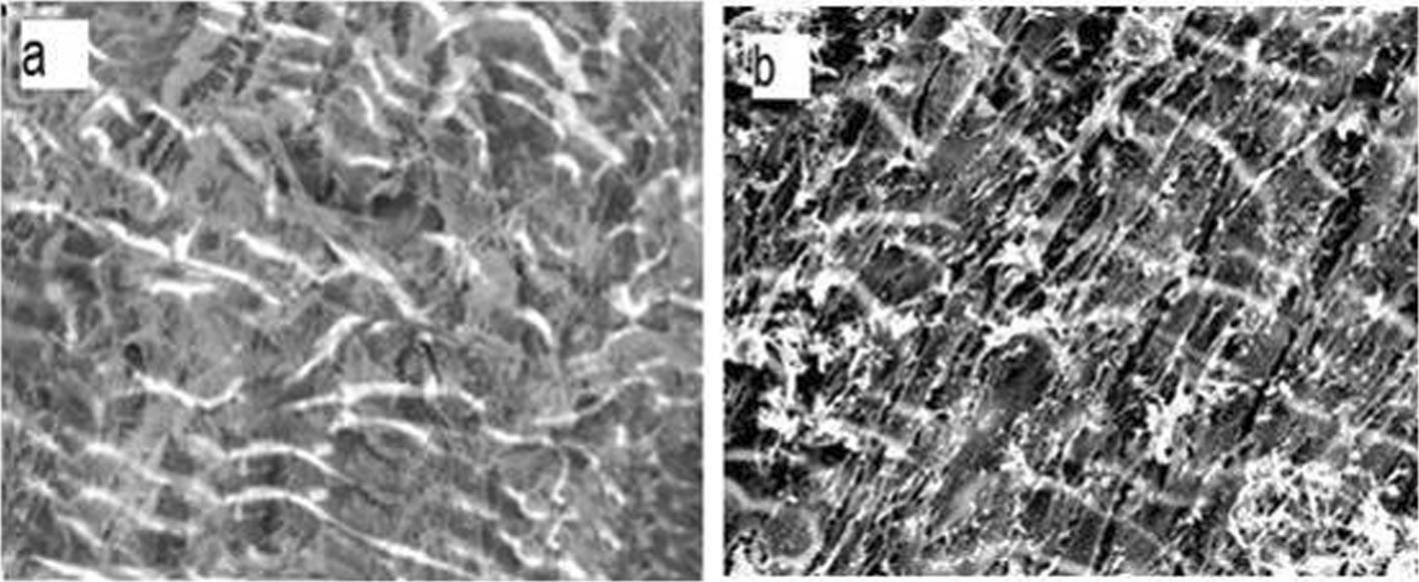
SEM images of **a** Saghez scaffold and **b** BMP2BMP2-loaded Saghez scaffold

**Fig. 5:**
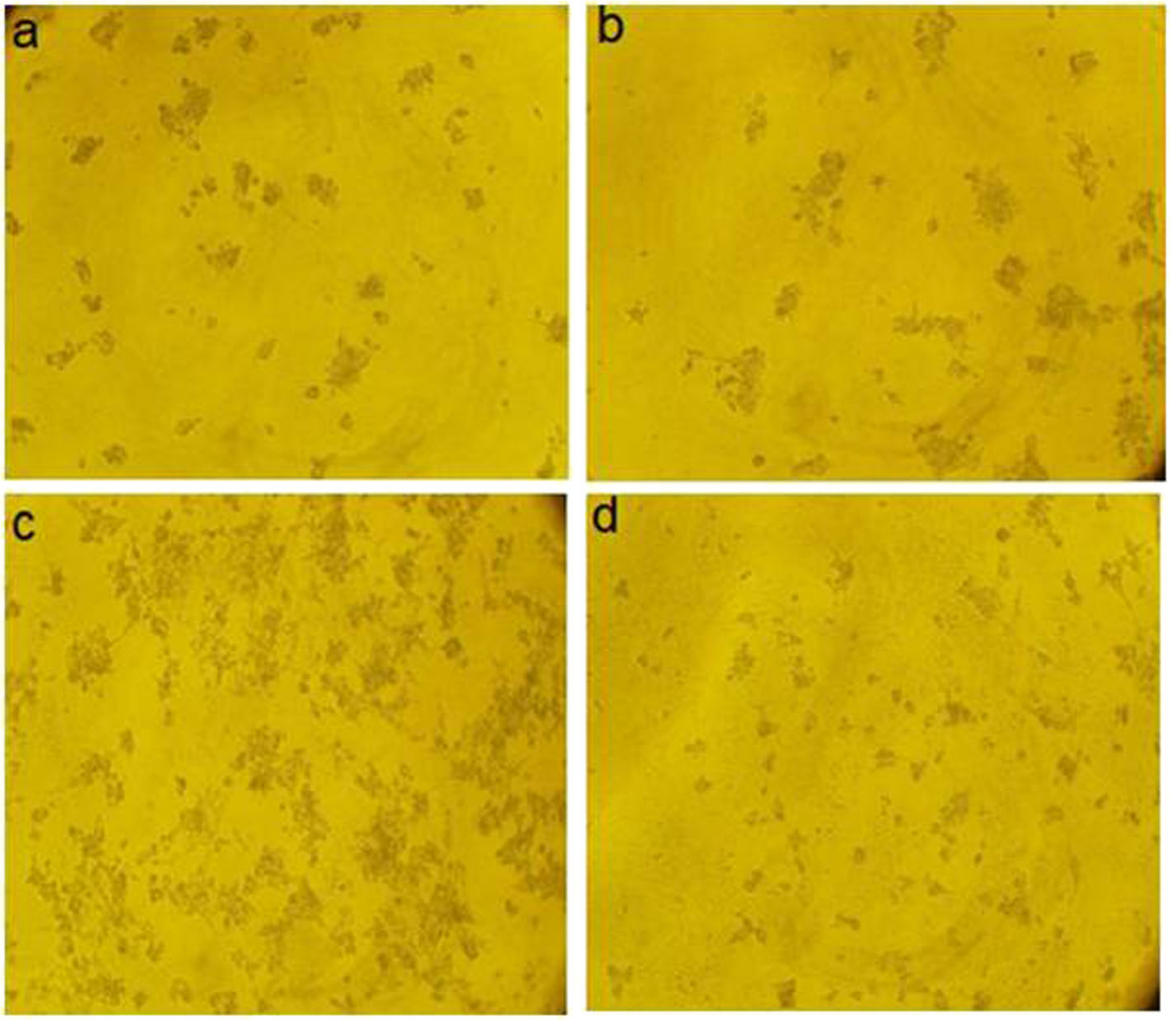
Morphological changes of the stem cells. **a**, **b** The morphology of the seeded cells in T75 culture flasks without differentiation medium. **c**, **d** The seeded cells cultured in the culture media containing differentiation agent

The cell were classified into four groups including scaffold alone, scaffold loaded with BMP2, cell culture containing differentiation factors, and cell culture without differentiation factors. SEM and phase contrast imaging were used to investigate morphological changes in the scaffold and cell culture groups, respectively. SEM imaging of Saghez scaffold illustrated that sufficient porosity and interconnection of pores, which are congruous for encapsulation of BMP2 and cell culture, were present in our fabricated scaffold (Fig. 4). No morphological changes were observed in cells treated with or without differentiation medium (Fig. 5.)

### Cell proliferation and viability

**Fig. 6:**
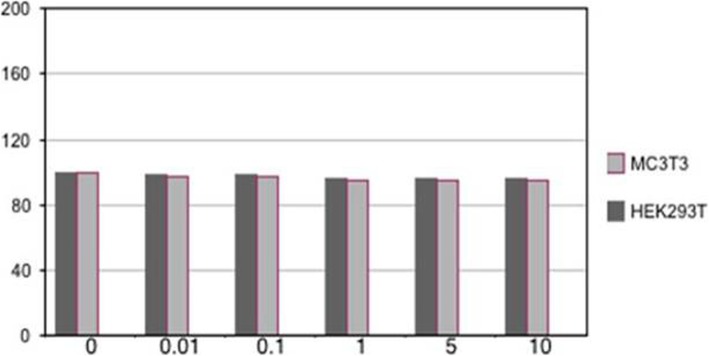
Viability rates of NT2 and HEK293T cell lines treated with Saghez scaffold for 72 h

MTT assay was applied to investigate the cytotoxicity of Saghez scaffold. The results of cell viability are presented in Fig. 5. The results indicated no significant differences between the viability rates of NT2 and HEK293T cell lines. The concentrations of 0.01 to 10 μg/μl of scaffold induced 4 to 6% and 3 to 5% cell death in NT2 and HEK293T, respectively (Fig. 6).

### In vitro drug release profile

**Fig. 7:**
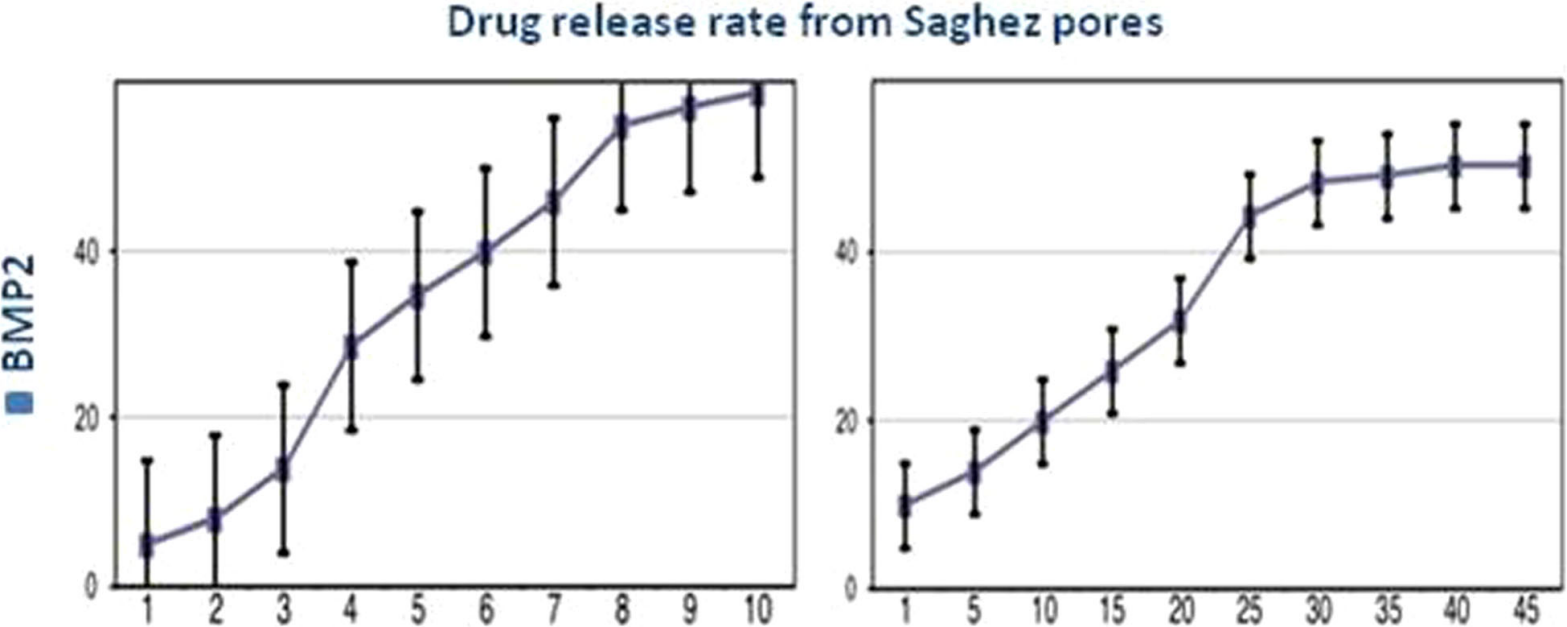
Drug release from Saghez scaffold

The release profile of BMP2 occurred in a sustained pattern without any burst release. Fifty percent of the encapsulated drug was released during the first 45 h. The gradual extrusion of BMP2BMP2BMP2 molecules extended for 45 h when the BMP2 release rate reached 96% (Fig. 7). The release of BMP2 molecules indicated the degradation of Saghez scaffold.

### Mechanical characteristics of Saghez scaffold

Mechanical properties of Saghez scaffold, including tensile Young’s modulus, ultimate tensile stress, compression Young’s modulus, and complex shear modulus were 19 MPa, 32 MPa, 0.42 MPa, and 0.9 MPa, respectively. The porosity of the scaffold was 70–140 μm, and the percentage of porosity was 75–98%.

### RT-PCR results and western blot analysis

**Fig. 8:**
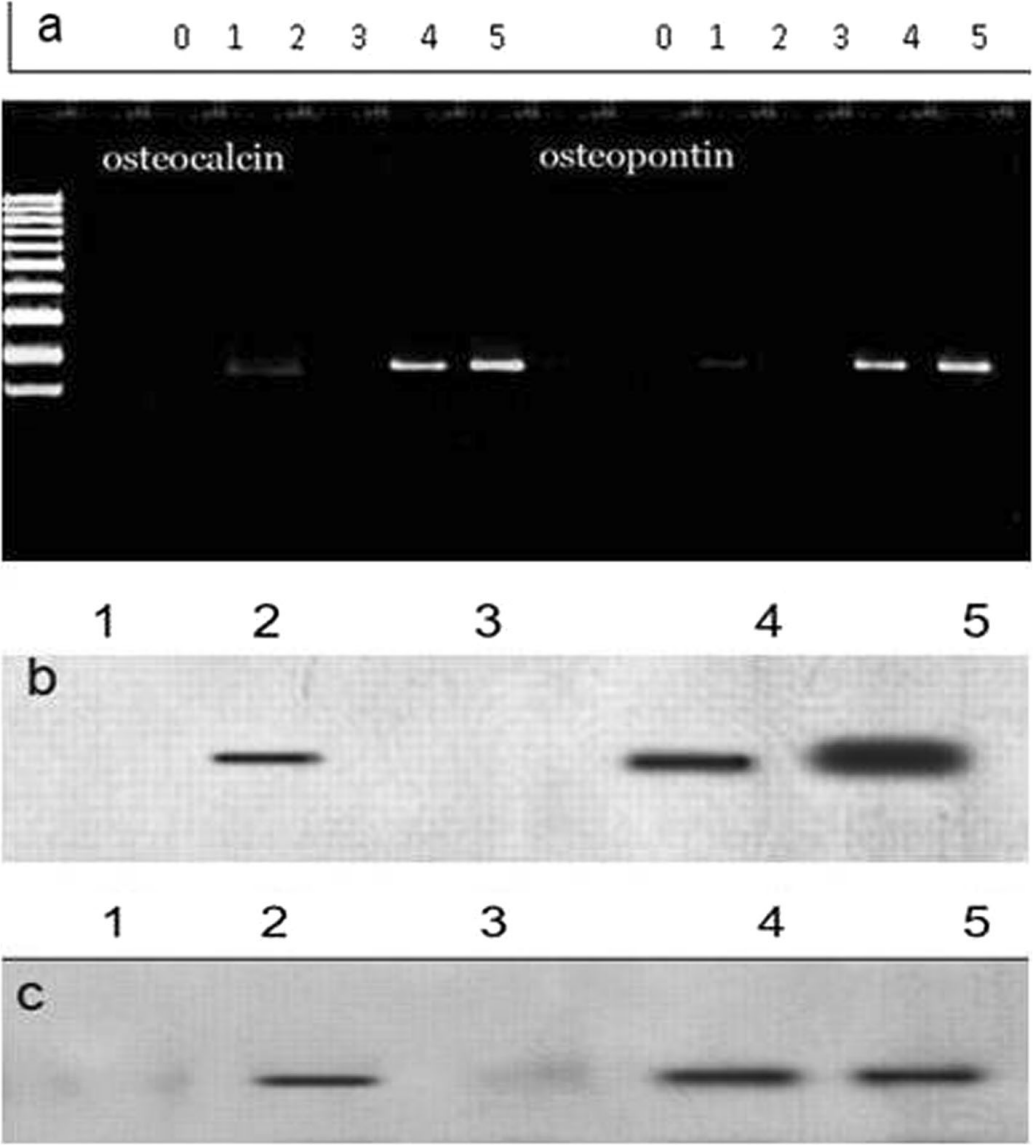
Results of the electrophoresis of the products of the PCR-based amplification of Osteopontin and osteocalcin (**a**) and protein expression profiles (**b** and **c**) in different experimental groups. **a**) Results of the electrophoresis of the products of the PCR amplification of Osteopontin and osteocalcin gene on day 21 post-cultured; 0: negative control (H2O), 1: Saghez scaffold without BMP2, 2: Saghez scaffold loaded with BMP2, 3: DMEM medium, 4: DMEM medium containing differentiation agents5: positive control

**Fig. 9:**
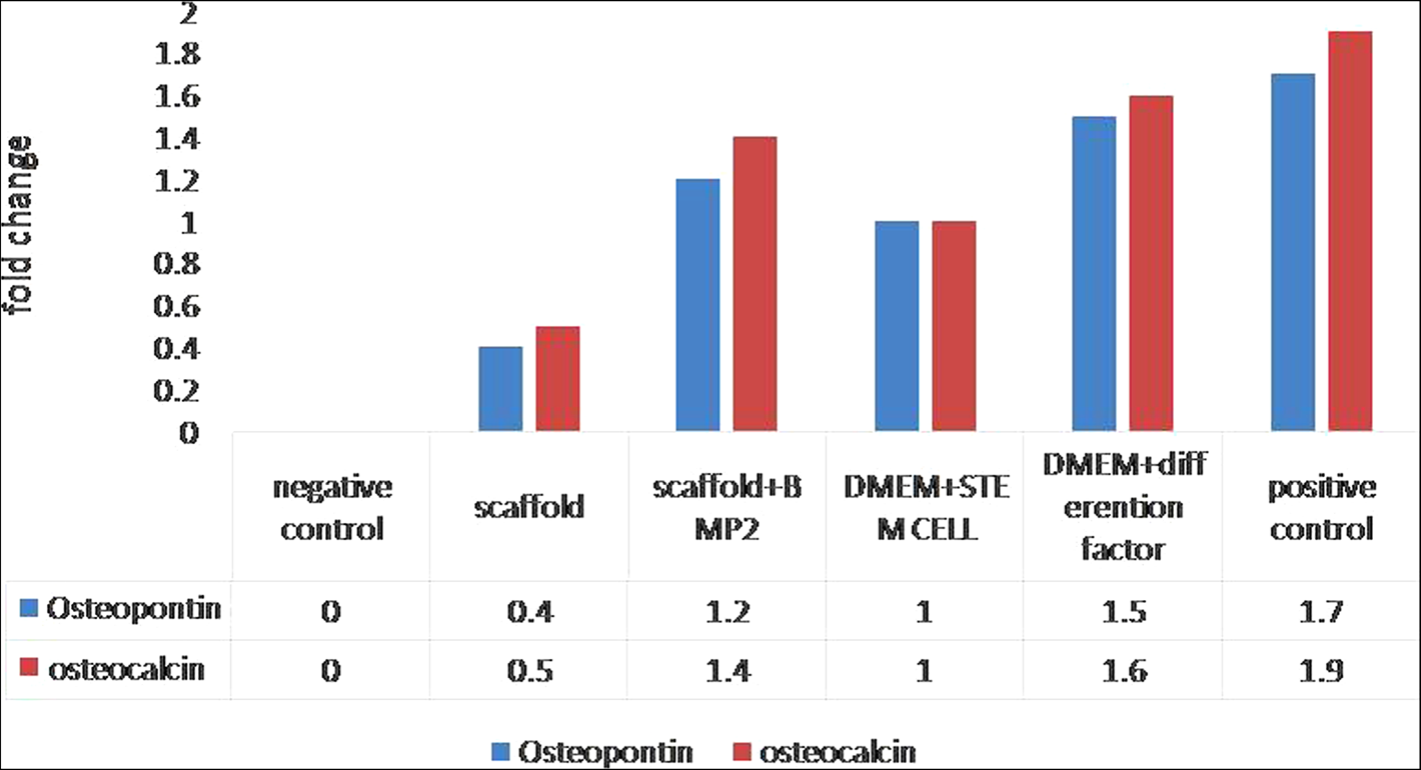
Gene expression fold change in various groups

The gene or protein expression levels of osteopontin and osteocalcin were drastically increased in the cells seeded on Saghez-BMP2 scaffold compared to those seeded on Saghez scaffold and the control cells cultures in medium without differentiation factor. However, the expression levels of both mentioned genes were the highest in the cells cultured in differentiation medium (Fig. 8). The highest osteopontin and osteocalcin gene expression levels (1.7 and 1.9) were seen in positive control, followed by DMEM + differentiation factor (1.5 and 1.6), scaffold + BMP2 (1.2 and 1.4), DMEM + stem cell (1 and 1) and scaffold (0.4 and 0.5), and negative control respectively (Fig. 9).

### Alizarin red and alkaline phosphatase assay

**Fig. 10:**
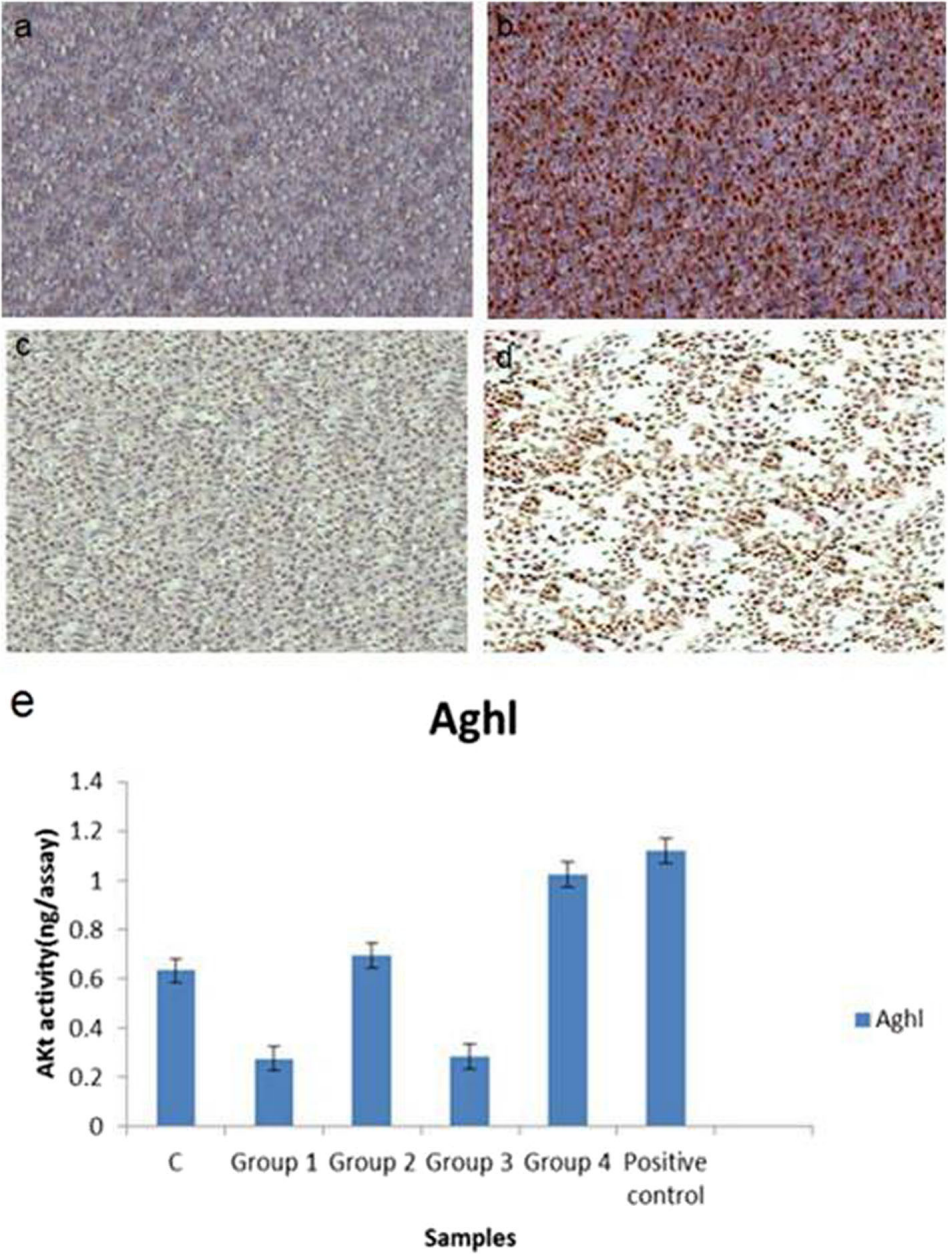
Alizarin staining of the cultured stem cells. **a** Saghez scaffold, **b** Saghez-BMP2 scaffold, **c** DMEM medium, **d** DMEM containing differentiation agent, and **e** histogram of alkaline phosphatase activity

The attachment of Alizarin to calcium granules of the mineralized cells indicates the presence of osteocyte cells. The results indicated that the rate of differentiation of the stem cells into osteocyte was the highest in the Saghez-BMP2 scaffold and differentiation medium groups (Fig. 10). Alkaline phosphatase activity was observed 21 days after culture. The highest level of enzyme activity was detected in differentiated cells cultured in differentiation medium compared to the other four groups. The second highest enzyme activity was related to the cells of Saghez-BMP2 scaffold. These differences were statistically significant (Fig. 10).

## Discussion

The loss of bone tissue is a prevalent issue in tooth extractions or during tumor surgeries. Therefore, bone regeneration is a required field in medicine. Recently, artificial bone preparation for face and mandible regeneration has been astonishingly progressed. Suitable scaffold, healthy stem cells, and differentiation factors are the three major issues that should be considered in tissue engineering. Therefore, synthesizing an efficient scaffold, particularly derived from natural sources with minimum reactions for implantation inside the body, is essential. In this study, stem cells were successfully isolated from three follicles of wisdom. This study represents the restoration of the natural regeneration of the bone defect. The scaffolds were synthesized using natural material, namely Saghez and BMP2 factor as a differentiating agent which was initially extracted from the bone. Auto-, allo-, and xenografts and bone graft substitutes have all been used to accelerate the healing of bone defects with some limitations. Bone allografts carry the risk of transmitting infectious and viral diseases, and also, they may stimulate immunological reactions [2]. Bone autografts have limitations in massive bone defects and pathologic fractures. A major concern with bone xenografts is the potential transmission of zoonotic diseases. Scaffolds are the most important issue in bone tissue engineering and divided into two main categories (natural or organic), and synthetic materials in which natural scaffolds have shown low antigenicity compared with others [2].

The wisdom tooth stem cells were differentiated into adipocytes and osteocyte, in the adipogenic and osteogenic differentiation media, respectively. These results are a potent proof for the presentation of stem cells in the isolated cells. The expression of the mesenchymal markers on the cell surface was a witness of the mesenchymal origin of extracted stem cells. Characterizations of the scaffold, including morphology, porosity, physical stability, in vitro drug release profile, and cytotoxicity, demonstrated that the synthesized scaffold could be used as an expedient tool to approach bone regeneration. Bone morphogenetic proteins strongly affect osteoblast differentiation. BMP2BMP2 is one of the renowned members of this family. It has been verified that BMP2BMP2 induces increment in Wnt1 and Wnt3a pathways that resulted in alkaline phosphatase enhancement [16]. Moreover, it is suggested that this agent is a key factor in bone grafts and replacement of bone tissue [17]. BMP2BMP2 is a homodimer protein whose receptors are expressed on the cell surface. BMP2 receptor-ligand binding affects intracellular pathways, including SMAD or MAPK, and activates other signaling pathways and regulatory proteins, such as Wnt, FDF, and Sox 9, respectively [18]. Giorgio et al. have reported that the extracted wisdom tooth follicle stem cells were differentiated to osteoblast. Exposition of mineralized nodules in the matrix and expression of alkaline phosphatase markers and collagen type1 triggered this differentiation [19]. In our study, the presence of mineralized nodules which was studied by Alizarin red assay and alkaline phosphatase expression indicated that the stem cell differentiated in both the scaffold containing BMP2BMP2 and osteogenic cell culture medium groups. In another study, HTGSCs [15] were isolated and the expression of substrates, such as oct4, sox2, and klf4, was assessed to specify the potency of the stem cells. The results of flow cytometric studies showed the presence of mesenchymal factors and verified that stem cells were differentiated into neurogenic, adipogenic, and osteogenic cells as the result of being treated with specific culture media. Tooth follicle is a useful tissue that surrounds the tooth and contains high amounts of mesenchymal stem cells. Unfortunately, these fruitful parts of the hidden tooth are extracted and discarded by dentists. Segregation and culture of these potent stem cells could be an exclamatory approach for bone and periodontal regeneration [20]. Huang et al. have compared BMMSCs [21] and tooth follicle-derived stem cells with regard to their potency in differentiation. BMMSCs are potent to differentiate into osteogenic, adipogenic, myogenic, neurogenic, and chondrogenic cells; however, the tooth-derived stem cells are more potent to differentiate into odontogenic cells, in contrast to BMMSCs [21].

Plant-derived resins have been widely applied in herbal medicine. Currently, the need for novel anti-bacterial substances is an indispensable requirement due to the development of resistance to first-line antibiotics. There is an assertion that plant-derived natural resins are vigorous compounds that act as anti-microbial agents with anti-bacterial, anti-fungal, and anti-protozoal activities [22]. It has been reported that Chios mastic gum acts as an anti-inflammatory and anti-oxidant substrate. Chios mastic is derived from *Pistacia lentiscus* var. chia and possesses anti-microbial activity; furthermore, it plays the gastrointestinal, cardiovascular, and hepatopreservator role. Moreover, Chios mastic is known to act as an anti-cancer material. It has been demonstrated that this herbal substrate reduces the superoxide and H_2_O_2_ contents by inhibiting protein kinase C and NADPH oxidase [12]. The cardiovascular and hepatic protectabilities of Chios have been verified by the diminution of LDL, serum total cholesterol, and the ratio of total cholesterol of serum to LDL after 5 mg of it was taken daily for 18 months [10]. This drug has been used for gastrointestinal treatment for more than 2500 years. It can overcome the growth of *H. pylori*. It has been shown that the activity of neutrophils was diminished in patients who are tested positive for *H. pylori*. These results were obtained after treating the patients with 1 g Chios, every day for 2 months [23]. Furthermore, it has been reported that Chios mastic gum plays an in vitro anti-tumoral role. It has been demonstrated that Chios and paclitaxel significantly reduce the proliferation rate of oral cancer cells. Both Chios and paclitaxel induce tumor cell apoptosis at the concentrations of 10 μg/ml and 50 μg/ml, respectively [11]. Saghez, similar to other plant-derived resins, is a widely used substance due to its remarkable exemplar characteristics, including anti-bacterial, anti-inflammatory, and anti-oxidant effects. Its extract acts as an anti-tumor and pro-apoptotic agent on YD-10B cell line [11]. The sol-gel method is an accepted procedure for preparation of a suitable scaffold. The porosity of a scaffold is one of its crucial properties since it affects the efficiency of cell seeding, migration, and adhesion. The results of MTT assay indicated that Saghez scaffold is not cytotoxic; therefore, it could be used as an expedient fabricated bone ECM. Isolation of the stem cells was a considerable part of this study. We followed the procedure presented by Bahrami et al. to isolate stem cells by digesting with the collagenase enzyme [24]. According to the mentioned study, all the stem cells were differentiated into osteocytes and adipocytes in their respective specific differentiation medium after 14 days. One of the important characteristics of a suitable scaffold is to maintain a loaded agent during a required period of time [14]. The release kinetics of BMP2 factor from Saghez scaffold was sustained and extended to 45 h; moreover, 96% of the loaded drug was released after 10 h which indicates the degradation of the scaffold. In the present study, stem cells were successfully differentiated into osteocytes on the Saghez-BMP2 matrix.

## Conclusion

In conclusion, our work provides a novel system for differentiation of the stem cell into osteocytes. The natural scaffolds display sufficient cytocompatibility and appropriate physical properties, such as porosity and strength, which make them suitable candidates for stem cell culture. The results of this study suggest that loaded BMP2 in Saghez scaffold possibly acts as an osteocyte differentiator factor.

## Data Availability

The datasets supporting the conclusions of this article are included within the article.

## Abbreviations

BMP2: Bone morphogenetic proteins
BMSCs: Bone marrow stem cells
FGF: Fibroblast growth factor
IGF: Insulin-like growth factor
PDGF: Periodontal ligament progenitor cells
PDLSCs: Adipose-derived stem cells and MSCs
TGF-β: Transforming growth factor-β
